# Transcriptome profiling analysis reveals that ATP6V0E2 is involved in the lysosomal activation by anlotinib

**DOI:** 10.1038/s41419-020-02904-0

**Published:** 2020-08-24

**Authors:** Xin Sun, Yuhan Shu, Peiyi Yan, Hongliang Huang, Ruilan Gao, Mengting Xu, Liqin Lu, Jingkui Tian, Dongsheng Huang, Jianbin Zhang

**Affiliations:** 1 Department of Oncology, Zhejiang Provincial People’s Hospital, People’s Hospital of Hangzhou Medical College, Hangzhou, China; 2 College of Biomedical Engineering & Instrument Science, Zhejiang University, Hangzhou, China; 3 Department of Clinical Laboratory, Shanghai Putuo District People’s Hospital, Shanghai, China; 4 School of Biosciences & Biopharmaceutics and Center for Bioresources & Drug Discovery, Guangdong Pharmaceutical University, Guangzhou, China; 5 Institution of Hematology Research, The First Affiliated Hospital of Zhejiang Chinese Medical University, Hangzhou, China; 6 Key Laboratory of Tumor Molecular Diagnosis and Individualized Medicine of Zhejiang Province, Clinical Research Institute, People’s Hospital of Hangzhou Medical College, Hangzhou, China

**Keywords:** Targeted therapies, Macroautophagy

## Abstract

Anlotinib is a receptor tyrosine kinase inhibitor with potential anti-neoplastic and anti-angiogenic activities. It has been approved for the treatment of non-small-cell lung cancer. Lysosomes are acidic organelles and have been implicated in various mechanisms of cancer therapeutics. However, the effect of anlotinib on lysosomal function has not been investigated. In the present study, anlotinib induces apoptosis in human colon cancer cells. Through transcriptome sequencing, we found for the first time that anlotinib treatment upregulates ATP6V0E2 (ATPase H^+^ Transporting V0 Subunit E2) and other lysosome-related genes expression in human colon cancer. In human colon cancer, we validated that anlotinib activates lysosomal function and enhances the fusion of autophagosomes and lysosomes. Moreover, anlotinib treatment is shown to inhibit mTOR (mammalian target of rapamycin) signaling and the activation of lysosomal function by anlotinib is mTOR dependent. Furthermore, anlotinib treatment activates TFEB, a key nuclear transcription factor that controls lysosome biogenesis and function. We found that anlotinib treatment promotes TFEB nuclear translocation and enhances its transcriptional activity. When TFEB or ATP6V0E2 are knocked down, the enhanced lysosomal function and autophagy by anlotinib are attenuated. Finally, inhibition of lysosomal function enhances anlotinib-induced cell death and tumor suppression, which may be attributed to high levels of ROS (reactive oxygen species). These findings suggest that the activation of lysosomal function protects against anlotinib-mediated cell apoptosis via regulating the cellular redox status. Taken together, our results provide novel insights into the regulatory mechanisms of anlotinib on lysosomes, and this information could facilitate the development of potential novel cancer therapeutic agents that inhibit lysosomal function.

## Introduction

Anlotinib, a new oral small-molecule receptor tyrosine kinase inhibitor developed by Chia-tai Tianqing Pharmaceutical Co., Ltd. in China, targets VEGFR1 (vascular endothelial growth factor receptor 1), VEGFR2, VEGFR3, c-Kit, PDGFR-α (platelet-derived growth factor receptor α), FGFR1 (fibroblast growth factor receptor 1), FGFR2, and FGFR3. Through these mechanisms, anlotinib can inhibit tumor angiogenesis and tumor cell proliferation^1,2^. The antitumor effect of anlotinib has been reported in many preclinical and clinical trials^3^. Compared to a placebo, anlotinib improved both progression-free survival and overall survival in a phase III trial in patients with advanced non-small-cell lung cancer^4,5^, despite progression of the cancer after two lines of prior treatments. Recently, anlotinib has been just approved as a third-line treatment for advanced non-small-cell lung cancer patients by the CFDA (China Food and Drug Administration). In addition, a randomized phase IIB trial in patients with advanced soft tissue sarcoma also demonstrates that anlotinib significantly extends the median progression-free survival^6,7^. In patients with advanced medullary thyroid carcinoma and metastatic renal cell carcinoma, anlotinib also shows promising efficacy^8–11^. In human colorectal cancer, the antitumor effect of anlotinib can be enhanced by MiR-940 targeting MACC1’s mRNA^12^. Similar to that of other receptor tyrosine kinase inhibitors, the combined treatment of anlotinib with chemotherapy does not appear to be more beneficial than anlotinib alone^13^.

Lysosomes are acidic organelles containing many degradative enzymes. At the late stage of autophagy, autophagosomes fuse with lysosomes and the contents of autophagosomes are degraded by lysosomal enzymes^14,15^. TFEB (transcription factor EB) is one of the most important molecular mechanisms regulating lysosomal function, and it is regulated mainly by its phosphorylation, which is mediated by the mTOR (mammalian target of rapamycin) kinase^16,17^. Our recent study^18^ revealed that TFEB is also regulated by another post-translational modification which is acetylation. Lysosomes play important roles in the tumorigenesis^19,20^, such as pancreatic and lung adenocarcinomas, which are characterized with mutant KRAS. The growth and survival of cancer cells in poor microenvironment demand for metabolites, which is achieved by systematically recycling both extracellular and intracellular components via scavenging pathways that converge on the lysosome^21^. Inhibition of lysosomal function can enhance the antitumor effect of many drugs^22–24^. Thus, targeting lysosomal function is emerging as a promising strategy in cancer therapeutics.

Compared with other tyrosine kinases, anlotinib occupies the ATP-binding pocket of VEGFR2 tyrosine kinase and shows high selectivity and inhibitory potency^1^. Upon anlotinib treatment, the downstream signaling pathways of VEGFR2 are inhibited, including ERK (extracellular regulated protein kinase) and AKT (protein kinase B) pathways^25^. It has been recently reported that anlotinib is able to induce autophagy, and autophagy inhibition enhances the cytotoxic effects of anlotinib and potentiates the anti-angiogenic property of anlotinib through JAK2 (Janus kinase 2)–STAT3 (signal transducer and activator of transcription 3)–VEGFA signaling^26^. But at the late stage of autophagy, the effect of anlotinib on lysosomal function has still been unknown.

Here, we hypothesize that anlotinib treatment activates lysosomal function and the biological function of lysosome in the anticancer effect of anlotinib is worth investigating. In this study, we found that anlotinib treatment leads to cell apoptosis in human colon cancer. Transcriptome sequencing analysis reveals that anlotinib targets ATP6V0E2 (ATPase H^+^ Transporting V0 Subunit E2) and other lysosome-related genes. We validated that anlotinib treatment activates lysosomal function via inhibiting mTOR signaling and enhancing TFEB transcriptional activity. TFEB or ATP6V0E2 knockdown attenuates anlotinib-induced lysosomal activation, which further enhances the cytotoxicity of anlotinib and leads to more cancer cell apoptosis. Taken together, our data support the notion that combined treatment with anlotinib and lysosomal inhibitors could be a novel promising therapeutic strategy in the treatment of colon cancer.

## Materials and methods

### Cell lines

HCT116 and SW480 cells were obtained from American Type Culture Collection. The GFP-LC3-expressing stable MEF cells, TSC2^+/+^ MEFs, TSC2^−/−^ MEFs, L929-tfLC3 cells were provided by Prof. Shen Han-Ming (National University of Singapore, Singapore). All cell lines were maintained in Dulbecco’s modified Eagle’s medium (DMEM) (Sigma-Aldrich, D1152) containing 10% fetal bovine serum (HyClone, SV30160.03) in a 5% CO_2_ atmosphere at 37 °C.

### Reagents

Antibodies were obtained as follows: anti-LC3 antibody (Sigma-Aldrich, L7543), anti-tubulin (Sigma-Aldrich, T6199), anti-FLAG (Sigma-Aldrich, F3165), anti-actin (Sigma-Aldrich, A5441), anti-TFEB (Bethyl Laboratories, A303-673A). All of the other antibodies were purchased from Cell Signaling Technology: anti-phospho-AKT (cata. no. 4060), anti-AKT (cata. no. 4691), anti-Bax (cata. no. 2774), anti-Bcl-2 (cata. no. 15071), anti-Cytochrome *c* (cata. no. 4272), anti-Caspase-3 (cata. no. 9662), anti-EGFR (cata. no. 2085), anti-GFP (cata. no. 2955), anti-Ki-67 (cata. no. 9027), anti-LAMP1 (cata. no. 9091S), anti-Lamin A/C (cata. no. 4777), phospho-mTOR (cata. no. 5536), anti-mTOR (cata. no. 2983), anti-phospho-S6 (cata. no. 2211), anti-S6 (cata. no. 2217), anti-PARP-1 (cata. no. 9542), anti-P62 (cata. no. 23214), anti-TSC2 (cata. no. 3612) and anti-14-3-3 (cata. no. 9638).

### Small interfering RNA (siRNA) and transient transfection

The scrambled RNAi oligonucleotides and siRNAs targeting TFEB (sc-38509; Santa Cruz Biotechnology) or ATP6V0E2 (GenePharma, Shanghai) were transfected into HCT116 cells using the Lipofectamine^®^ 3000 according to the manufacturer’s protocol. After 48 h, the cells were subjected to the designated treatment. For plasmid transfection, cells were transiently transfected with GFP-TFEB or FLAG-TFEB plasmids using the Lipofectamine^®^ 2000 according to the manufacturer’s protocol. Plasmids were kindly provided by Prof. Shen Han-Ming (National University of Singapore, Singapore) as described^18,27^.

### LysoTracker staining

After the designated treatments, cells were incubated with 50 nM LysoTracker Red in DMEM for 30 min for labeling and tracking acidic organelles in live cells. The cells in the chambered coverglass were observed under a confocal microscope.

### Magic Red cathepsin B and L activity assay

Lysosomal function was also estimated by the cathepsin B and L enzymatic activity. After designated treatment, cells were further loaded with Magic Red^TM^ cathepsin B (Immunochemistry Technologies, 938) or cathepsin L (Immunochemistry Technologies, 942) reagents for 30 min. The cells were collected and the fluorescence intensities of 10,000 cells per sample were measured by flow cytometry. We recorded the fluorescence of Magic Red using the FL-2 channel of FACS (BD Biosciences).

### Confocal imaging

Cells were first cultured on eight-well Lab-Tek^TM^ chambered coverglass (Thermo Scientific, 155411) overnight, followed by designated treatment. All of the confocal images were obtained with ×60 oil objective (numerical aperture 1.4) lenses of Leika TCS SP5 Confocal.

### Measurement of ROS production

CM-H_2_DCFDA (Invitrogen, C6827) was chosen for the detection of intracellular ROS production. After the designated treatments, cells were incubated with 1 μM CM-H_2_DCFDA in phosphate-buffered saline (PBS) for 10 min. Then cells were collected and fluorescence intensity was measured. We recorded the fluorescence of CM-DCF using the FL-1 channel of FACS (BD Biosciences).

### Western blotting

After the indicated time of designated treatment, cells were collected and rinsed with PBS. The whole-cell lysates were prepared in the Laemmli buffer (62.5 mM Tris-HCl, pH 6.8, 20% glycerol, 2% sodium dodecyl sulfate (SDS), 2 mM DTT, phosphatase inhibitor, and proteinase inhibitor mixture). An equal amount of protein was resolved by SDS-PAGE and transferred onto PVDF membrane. After blocking with 5% nonfat milk, the membrane was probed with designated first and second antibodies, developed with the enhanced chemiluminescence method (Thermo Scientific, 34076), and visualized using the Bio-Rad ChemiDoc MP Imaging System.

### Luciferase assay

TFEB luciferase vector was provided by Prof. Shen Han-Ming (National University of Singapore). The transient transfection of the TFEB luciferase vector was done in HCT116 cells using the Lipofectamine^TM^ 2000 transfection reagent according to the manufacturer’s protocol. Renilla luciferase vector was used as a transfection control. The luciferase activity was measured at 48-h time after transfection using the Dual-Luciferase reporter assay system (Promega, E1960) based on the protocol provided by the manufacturer.

### Reverse transcription and quantitative real-time PCR

RNA was extracted with the RNeasy kit (Qiagen, 217004). A reverse transcription reaction was performed using 1 μg of total RNA with iScript^TM^ Reverse Transcription Supermix for quantitative reverse transcription PCR (RT-qPCR) (Bio-Rad, 170-8841). The mRNA expression levels were determined by real-time PCR using SsoFast EvaGreen Supermix (Bio-Rad, 172-5201AP) and the CFX96 Touch Real-time PCR Detection System (Bio-Rad). Glyceraldehyde-3-phosphate dehydrogenase was used as an internal control of RNA integrity. Real-time PCR was performed in triplicate.

### Transcriptome sequencing

Total RNA was isolated and purified using TRIzol reagent (Invitrogen, Carlsbad, CA, USA) following the manufacturer’s procedure. The RNA amount and purity of each sample was quantified using NanoDrop ND-1000 (NanoDrop, Wilmington, DE, USA). The RNA integrity was assessed by Agilent 2100 with RIN number >7.0. Poly(A) RNA is purified from total RNA(5 μg) using poly-T oligo-attached magnetic beads using two rounds of purification. Then the poly(A) RNA was fragmented into small pieces using divalent cations under high temperature. Then the cleaved RNA fragments were reverse-transcribed to create the cDNA, which were next used to synthesize U-labeled second-stranded DNAs with *Escherichia coli* DNA polymerase I, RNase H and dUTP. An A-base is then added to the blunt ends of each strand, preparing them for ligation to the indexed adapters. Each adapter contains a T-base overhang for ligating the adapter to the A-tailed fragmented DNA. Single- or dual-index adapters are ligated to the fragments, and size selection was performed with AMPureXP beads. After the heat-labile UDG enzyme treatment of the U-labeled second-stranded DNAs, the ligated products are amplified with PCR by the following conditions: initial denaturation at 95 °C for 3 min; 8 cycles of denaturation at 98 °C for 15 s, annealing at 60 °C for 15 s, and extension at 72 °C for 30 s; and then final extension at 72 °C for 5 min. The average insert size for the final cDNA library was 300 bp (±50 bp). At last, we performed the 150-bp paired-end sequencing on an Illumina X Ten (LC Bio, China) following the vendor’s recommended protocol.

### Colony formation assay

HCT116 cells (200 cells) were cultured in six-well plate for 48 h and then treated with different doses of anlotinib. After that, cells were cultured for 12–20 days in culture medium. Surviving colonies were stained with gentian violet after methanol fixation and visible colonies (≥50 cells) were counted. The experiments were performed in triplicate.

### Cell proliferation and cytotoxicity assay

Cells were seeded in a 96-well plate and treated with anlotinib in the presence or absence of bafilomycin for 24 h. After treatment, 10 μl of CCK-8 (cell counting kit 8, E606335-0500; Sangon Biotech, Shanghai) solution was added to each well of the plate and incubated the plate for 1–4 h in the incubator. Finally, the absorbance at 450 nm was measured using a microplate reader.

### Detection of cell death

Cell death was estimated by morphological changes under phase-contrast microscopy and quantified by Pacific Blue™ Annexin V (A35122, Thermo Fisher Scientific) and PI (propidium iodide, V13244; Thermo Fisher Scientific) staining coupled with flow cytometry (BD Biosciences). Western blotting was also used to indicate the cell death via PARP-1 and Caspase-3 cleavages.

### In vivo xenograft tumor model

Four-week-old male BALB/c nude mice were purchased from the Institute of Zoology, Zhejiang Chinese Medical University. All experiments were performed in accordance with the official recommendations of the Chinese Zoological Society, and animals received humane care according to the criteria outlined in the “Guide for the Care and Use of Laboratory Animals”. A suspension containing 3 × 10^6^ HCT116 cells was subcutaneously injected into the right flanks of the nude mice. After 10 days, all mice were randomly divided into four groups: control; anlotinib 50 mg/kg; BAF, 1 mg/kg; anlotinib plus bafilomycin. The tumor dimensions were measured using a Vernier caliper twice per week. Mice were killed 30 days after inoculation, and xenograft tumors were weighed.

### Statistical analysis

All western blotting data and image data presented are representative of three independent experiments. The numeric data except for RT-qPCR data are presented as mean ± SD from three independent experiments and analyzed using Student’s *t*-test.

## Results

### Anlotinib inhibits cell growth and induces apoptosis in human colon cancer cells

**Fig. 1:**
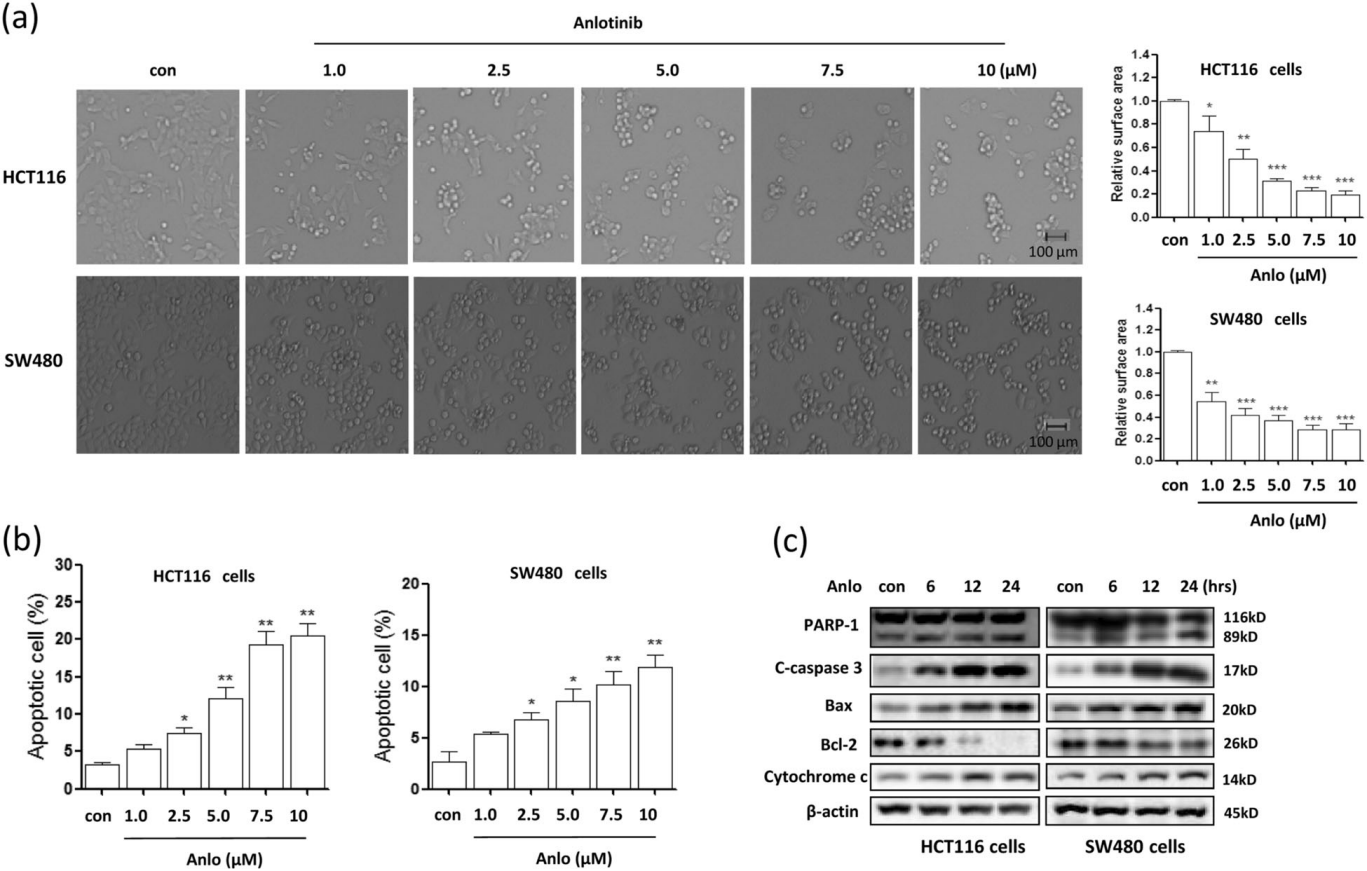
Anlotinib inhibits cell growth and induces apoptosis in a time- and dose-dependent manner in human colon cancer cells. **a** HCT116 and SW480 cells were treated with anlotinib (1–10 μM) for 24 h as indicated. Changes in cell morphology following each treatment condition were examined and captured with an inverted microscope (scale bar: 100 μm). **b** As in **a**, cells were stained using Annexin V-Pacific blue and PI, and cell fluorescence was detected with flow cytometry. The data are presented as the mean ± SD (*P < 0.05, **P < 0.01). **c** HCT116 or SW480 cells were treated with 5 μM anlotinib for different time and harvested. The whole-cell lysates were prepared and subjected to western blotting analysis using antibodies against PARP-1, cleaved-caspase-3, Bax, Bcl-2 and Cytochrome *c*. β-actin was used as a loading control.

Anti-angiogenesis agents exert their antitumor effect by reducing tumor microvascular density and increasing tumor hypoxia^28^. We first evaluated the effect of anlotinib on cell growth in human HCT116 and SW480 colon cancer cells. As shown in Supplementary Fig. 1, we observed a significant decrease in both colony size and number with increasing doses. At a concentration of 2.5 μM, there was an 80% reduction in colony numbers. Next, we determined cell death changes in anlotinib-treated human colon cancer cells. As shown in Fig. 1a, cell morphology changes showed the shrinked, round and low-density cells under anlotinib treatment and more cell death were observed as the doses increased from 1.0 to 10 μM, indicating a dose-dependent cytotoxic effect. We next analyzed apoptosis using annexin V and PI staining to quantify apoptotic cells and found that anlotinib significantly induced cell apoptosis at the concentration of 2.5–10 μM in HCT116 cells or SW480 cells (Fig. 1b and Supplementary Fig. 2a, b). We also investigated whether anlotinib-induced cell death was associated with treatment time. HCT116 or SW480 cells were treated with anlotinib for different time, and we observed that anlotinib treatment led to more cell apoptosis with the time increased (Supplementary Fig. 2c), suggesting a time-dependent effect. Moreover, our western blotting analysis also revealed that anlotinib increased the expression of the pro-apoptotic proteins, including the cleaved PARP-1 and Caspase-3, Bax and Cytochrome *c*, but decreased the expression of anti-apoptotic protein Bcl-2 (Fig. 1c).

### Identification of ATP6V0E2 as anlotinib target through transcriptome sequencing

To identify potential anlotinib targets, we performed a transcriptome sequencing analysis and screened a large number of genes in human HCT116 cells treated with anlotinib. Compared with the control group, the expression levels of 283 genes were significantly altered following anlotinib treatment. Among these differentially expressed genes, 116 genes were downregulated, and 167 genes were upregulated.

**Fig. 2:**
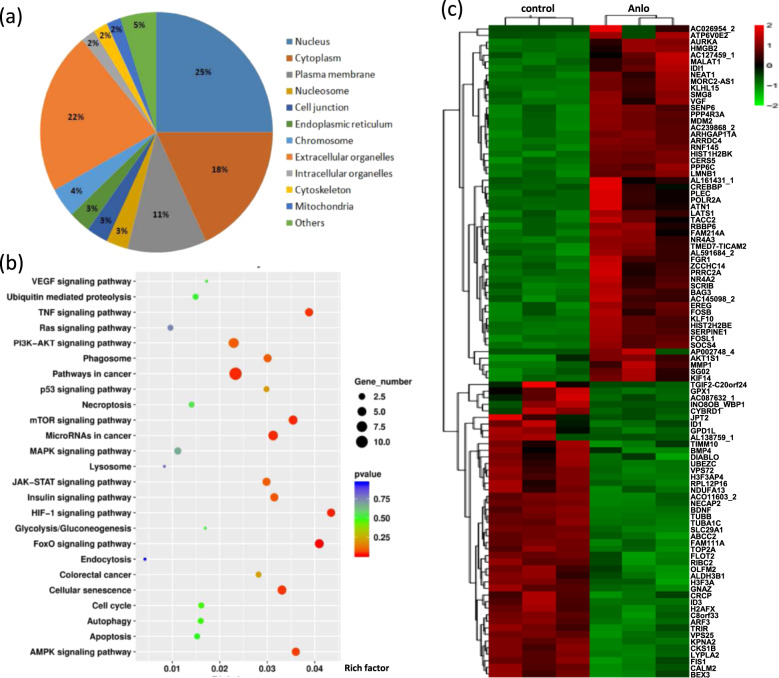
Anlotinib exerts an inhibitory effect on lysosome-related signaling. HCT116 cells were treated with anlotinib (2.5 μM, 12 h) and harvested for RNA extraction. Transcriptome sequencing was performed for human gene expression analysis. **a** GO analysis of cellular localization of the anlotinib targets. **b** KEGG enrichment of signaling pathways in anlotinib-treated cells. The dot represents gene number. **c** Effect of anlotinib on expression of target genes in HCT116 cells. Red and blue represent upregulated and downregulated genes, respectively. The fold changes were shown on the top-right corner.

Subsequently, we performed GO (gene ontology) analysis of anlotinib targets. It was shown that these targets are broadly distributed in different parts of the cell, and especially enriched in the nucleus, cytoplasm and plasma membrane (Fig. 2a and Supplementary Table 1). KEGG (kyoto encyclopedia of genes and genomes) analysis showed that anlotinib targets were involved in various signaling pathways, including FoxO (forkhead box), mTOR, AMPK (AMP-activated protein kinase), insulin, Jak–STAT (Janus kinase–signal transducer and activator of transcription), PI3K (phosphoinositide 3-kinase)–AKT, p53, TNF (tumor necrosis factor), VEGF, MAPK (mitogen-activated protein kinase), Ras, Wnt, HIF (hypoxia inducible factor) and so on (Fig. 2b). These signaling pathways were associated with autophagy, lysosome, phagosome, endocytosis, proteolysis, glycolysis, gluconeogenesis, cell cycle, apoptosis, necroptosis and so on. Among them, the apoptosis and oxidative stress pathways, such as FoxO, p53, TNF and HIF were activated with the upregulation of series of genes, including SERPINE1 (serpin family e member 1), MDM2 (murine double minute 2), GRB2 (growth factor receptor bound protein 2), CREBBP (CREB-binding protein), CREB3L3 (CAMP responsive element-binding protein 3 like 3) and CXCL1 (C-X-C motif chemokine ligand 1). In contrast, the cell proliferation pathways, such as PI3K–AKT, mTOR, AMPK, VEGF, Wnt, MAPK and Ras, were significantly suppressed with the downregulation of series of genes, including ITGA2 (integrin subunit alpha 2), AKT2, AKT1S1 (AKT1 substrate 1), PIK3R2, PIK3R5, WNT2B, WNT4, HNF4A (hepatocyte nuclear factor 4 alpha), CACNA1I (calcium voltage-gated channel subunit alpha1 I), SOCS2 (suppressor of cytokine signaling 2), ENO4 (enolase 4) (Supplementary Fig. 3). It was consistent with the activation of apoptosis and the inhibition of cell growth by anlotinib in human colon cancer (Fig. 1).

It has been well known^16^ that the lysosome senses its content and regulates its own biogenesis by a lysosome-to-nucleus signaling mechanism that involves PI3K–AKT and mTOR signaling pathways. TFEB, a master regulator of lysosomal biogenesis, is phosphorylated by mTOR and transcriptionally regulates lysosome-related genes expression^29^. Our transcriptome sequencing analysis demonstrated that the PI3K–AKT and mTOR signaling pathways were inhibited, which involved CREB3L3, GRB2, ITGA2, ITGB8, MDM2, PIK3R2, PIK3R5, WNT2B and WNT4 genes (Supplementary Fig. 3 and Supplementary Table 2). As a result, the formation of phagosome was enhanced with the upregulation of series of genes, including ATP6V0E2, ATP6V1C2, ATP6V1E2, CTSW (cathepsin W), CTSK (cathepsin K), IRS2 (insulin receptor substrate 2), which were also TFEB target genes.

In the significantly altered genes, 55 upregulated genes and 44 downregulated genes were displayed (Fig. 2c). Among them, ATP6V0E2 was identified as a novel downstream target of anlotinib and also the most significant change (Supplementary Table 3). Here, it was chosen for further study. H^+^-ATPases acidify various intracellular compartments, such as lysosomes. Multiple subunits form H^+^-ATPases, with proteins of the V1 class hydrolyzing ATP for energy to transport H^+^, and proteins of the V0 class forming an integral membrane domain through which H^+^ is transported. ATP6V0E2 encodes an isoform of the H^+^-ATPase V0 e subunit, an essential proton pump component^30^. Other H^+^-ATPase subunits were also upregulated by anlotinib, such as ATP6V1C2 and ATP6V1E2 (Supplementary Fig. 3). In addition, the expression levels of cathepsins were also increased, which are ubiquitously expressed lysosomal aspartyl protease and involved in the normal degradation of proteins, including CTSW and CTSK. All of them were required for the formation of phagosome.

### Anlotinib activates lysosomal function in human colon cancer cells

**Fig. 3:**
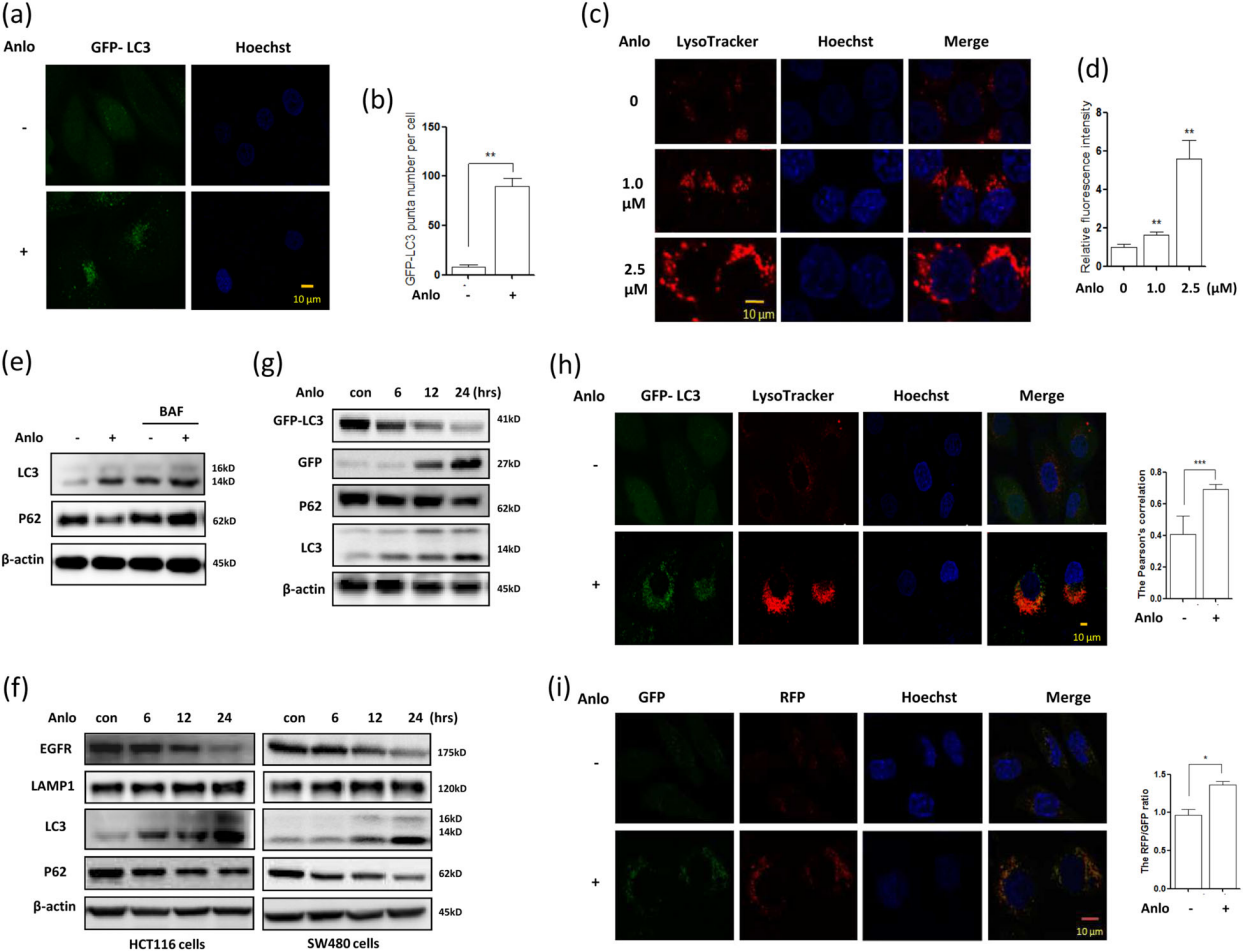
Anlotinib activates lysosomal function and enhances the fusion of autophagosomes and lysosomes in human colon cancer cells. **a**, **b** MEFs stably expressing GFP-LC3 were treated with anlotinib (2.5 μM) for 12 h. GFP-LC3 puncta were then examined by confocal microscopy (scale bar: 10 μm) and statistically analyzed using Student’s *t*-test (**P < 0.01). **c**, **d** As in **a**, HCT116 cells were treated with anlotinib followed by loading with LysoTracker Red (50 nM) for 15 min. Fluorescence intensity was measured with a confocal microscope (scale bar: 10 μm) and statistically analyzed using Student’s *t*-test (**P < 0.01). **e** HCT116 cells were tr**e**ated with anlotinib (2.5 μM) with or without bafilomycin for 12 h. The cells were then harvested for western blotting to examine LC3 and P62 levels. β-actin served as a loading control. **f** HCT116 and SW480 cells were treated with anlotinib (2.5 μM) for different times (6, 12 or 24 h). Cell lysates were prepared for western blotting to determine autophagy- and lysosome-related protein levels. β-actin was used as a loading control. **g** As in **a**, MEFs stably expressing GFP-LC3 were treated with anlotinib for different time. GFP-LC3 cleavage was determined using western blotting. **h** MEF cells stably expressing GFP-LC3 were treated with anlotinib (2.5 μM, 12 h). After staining with LysoTracker, cells were examined by confocal microscopy (scale bar 10 μm). Their colocalization was also analyzed using ImageJ and statistically analyzed using Student’s *t*-test (***P < 0.001). **i** Anlotinib increased the RFP signal in the L929-tfLC3 cells. Cells were treated with anlotinib for 12 h and then examined under a confocal microscope (scale bar 10 μm). The ratio of RFP to GFP was also calculated and statistically analyzed using Student’s *t*-test (*P < 0.05).

We next sought to examine the effect of anlotinib treatment on the lysosomal function. As shown in Fig. 3a, b, anlotinib significantly increased the formation of GFP-LC3 puncta (a marker of autophagosome formation) in MEFs in a dose-dependent manner. In human colon cancer HCT116 cells, LysoTracker staining showed that lysosomal acidification was significantly increased with following treatment (Fig. 3c, d). Moreover, we determined the autophagy flux level and found that anlotinib treatment further increased LC3-II levels in the presence of BAF (bafilomycin A1) (Fig. 3e and Supplementary Fig. 4), a known autophagy inhibitor blocking V-ATPase activity. The levels of autophagy substrate P62 were reduced by anlotinib treatment, indicating the increased autophagy flux.

In addition, we treated HCT116 or SW480 cells with anlotinib for different time and observed that the levels of LC3 (autophagosome marker) and LAMP1 (lysosome-associated membrane protein 1) increased over time while P62 levels decreased accordingly (Fig. 3f). EGFR (epidermal growth factor receptor) is known to be degraded by the lysosome and we measured its changes in abundance in anlotinib-treated cells. As shown in Fig. 3f, anlotinib treatment induced time-dependent EGFR degradation in HCT116 and SW480 cells. Because GFP-LC3 degradation occurs within autolysosomes^31^, we also examined free GFP fragments, which reflect the lysosomal degradation. As shown in Fig. 3g, anlotinib treatment increased the levels of free GFP fragments; in contrast, the levels of GFP-LC3 decreased over time. These results indicate the enhanced lysosomal degradation.

During the late stage of autophagy, autophagosomes fuse with lysosomes and are subsequently degraded^27^. Here, we also determined the effect of anlotinib on the fusion of autophagosomes and lysosomes. As shown in Fig. 3h, in MEFs stably expressing GFP-LC3, anlotinib treatment significantly increased the formation of GFP-LC3 puncta in the lysosomes. Moreover, we used L929 cells stably expressing tfLC3 (mRFP-GFP tandem fluorescent-tagged LC3) to examine their fusion^32^. As shown in Fig. 3i, we observed that the RFP-LC3 puncta increased more than the GFP-LC3 puncta following anlotinib treatment. The above findings demonstrate that anlotinib activates lysosomal function by promoting autophagosome–lysosome fusion.

### The activation of lysosome by anlotinib is due to mTOR suppression

**Fig. 4:**
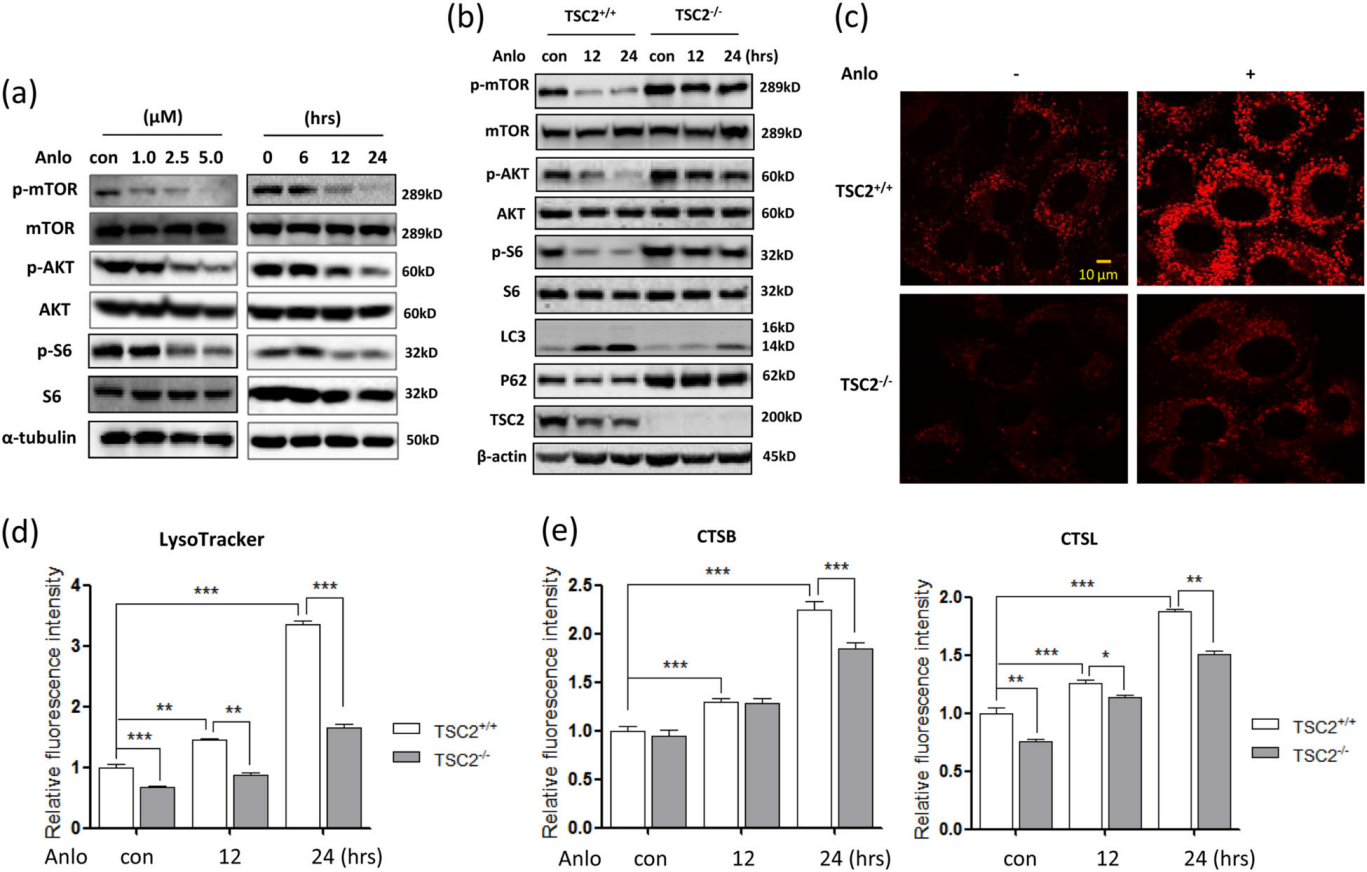
Anlotinib activates lysosomal function in a mTOR-dependent manner. **a** HCT116 cells were treated with different doses of anlotinib (1.0, 2.5 or 5 µM, left panel) or treated with anlotinib (2.5 µM) for different times (6, 12 or 24 h, right panel) as indicated. Cells were harvested and lysed for western blotting to determine phospho-AKT (Ser473) and phospho-S6 (Ser235/236) levels. β-actin was used as a loading control. **b** TSC2^+/+^ and TSC2^−^^/−^ MEFs were treated with anlotinib (2.5 µM) for 12 or 24 h. After the designated treatment, cells were harvested and cell lysates were prepared for western blotting to examine the levels of phospho-mTOR (Ser2448), phospho-AKT (Ser473) and phospho-S6 (Ser235/236). Levels of the autophagy markers LC3-II and P62 were also determined. β-actin was used as a loading control. **c**, **d** As in **b**, after designated treatment, cells were stained with LysoTracker Red (50 nM, 15 min). Cell fluorescence was measured by confocal microscopy (scale bar: 10 μm), and quantified by flow cytometry. **e** As in **b**, the treated cells were loaded with Magic Red for cathepsin B or cathepsin L for 15 min. Flow cytometry was performed to determine cell fluorescence, and the values were calculated and statistically analyzed using Student’s *t-*test (*P < 0.05, **P < 0.01, ***P < 0.001).

One of the most important molecular mechanisms in the regulation of lysosomal function in the course of autophagy is mTOR signaling^16,27^. Here, we examined the effect of anlotinib on mTOR activity. As shown in Fig. 4a and Supplementary Fig. 5, anlotinib treatment decreased phospho-AKT, -mTOR and -S6 levels in HCT116 or SW480 cells in a time- and dose-dependent manner, indicating the suppression of the AKT–mTOR signaling pathway.

To further establish the role of mTOR in regulating lysosomal function, we utilized TSC2^−/−^ MEFs with constitutively active mTOR^33^. Western blotting results showed that the basal activity of mTOR was higher in TSC2^-/-^ cells than in TSC2^+/+^ cells (Fig. 4b). Anlotinib treatment for 12 h decreased the levels of phospho-mTOR, -AKT and -S6. In TSC2^−^^/−^ cells, however, anlotinib treatment failed to suppress mTOR activity. Consequently, anlotinib induced autophagy in TSC2^+/+^ cells as LC3 levels increased and P62 levels decreased over time, but these changes did not occur in TSC2^−/−^ cells (Fig. 4b).

LysoTracker and AO (acridine orange) staining show that TSC2^+/+^ cells had a higher basal level of lysosomal acidification and anlotinib treatment increased the lysosomal acidification to a greater extent in TSC2^+/+^ cells compared with TSC2^−^^/−^ cells (Fig. 4c). Flow cytometry was performed to quantify the changes of cell fluorescence in this set of MEFs and cell fluorescence was significantly increased in TSC2^+/+^ cells over time under anlotinib treatment (Fig. 4d). When compared with that in TSC2^−/−^ cells, the increase in cell fluorescence in TSC2^+/+^ cells was far greater (Fig. 4d). In addition, lysosomal enzyme activity, such as cathepsin B and cathepsin L, was also determined using Magic Red staining. As shown in Fig. 4e, anlotinib treatment led to a more significant increase in cathepsin B and L activity over time in TSC2^+/+^ cells compared with that in TSC2^−^^/−^ cells. These results thus indicate that lysosomal activation by anlotinib is most likely mediated by its suppressive effect on mTOR activity.

### Anlotinib treatment increases TFEB transcriptional activity

**Fig. 5:**
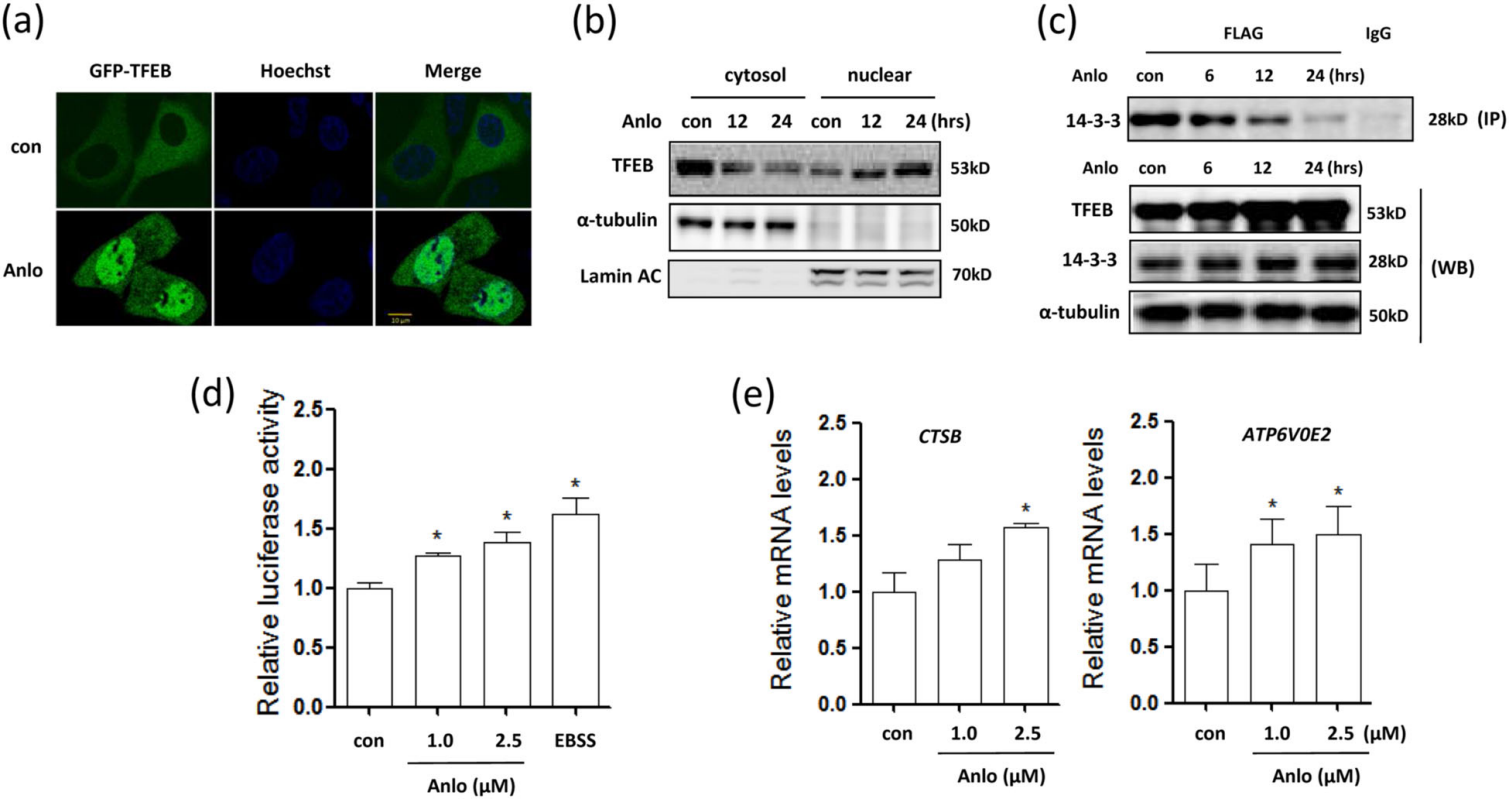
Anlotinib enhances TFEB transcriptional activity. **a** Anlotinib treatment promoted TFEB nuclear translocation (2.5 µM, 12 h). Live-cell imaging of GFP-TFEB (green) and Hoechst (blue) staining in HEK293 cells showed an enrichment of the GFP-TFEB signal in the nucleus (scale bar: 10 μm). **b** As in **a**, cells were treated with 2.5 μM anlotinib for different times (6 or 12 h) as indicated. To track the subcellular localization of TFEB, nuclear and cytosolic proteins from control and anlotinib-treated cells were probed for TFEB. The same membrane was then stripped and reprobed for α-tubulin or lamin A/C to ensure equal protein loading. **c** HEK293T cells were transiently transfected with TFEB-3x FLAG and then treated with 2.5 μM anlotinib for different times. The cells were lysed and subjected to immunoprecipitation with an anti-FLAG antibody, followed by immunoblotting for 14-3-3. TFEB was also determined using an anti-FLAG antibody. **d** The TFEB luciferase reporter construct was first transiently transfected into HCT116 cells, which were then treated with anlotinib (1.0 or 2.5 μM) for 12 h. The relative luciferase activity was measured using a Dual-Luciferase^®^ Reporter assay. The error bars represent the standard deviation from two independent experiments. Cells subjected to EBSS starvation are also shown. **e** HCT116 cells were treated with anlotinib (1.0 or 2.5 μM; 12 h) and then harvested for RNA extraction. Changes in the mRNA levels of some known TFEB target genes were measured using real-time PCR. GAPDH served as an internal control. All values are the means ± SD for at least three independent experiments. Student’s *t*-test (*P < 0.05).

TFEB transcriptionally regulates the expression of autophagy- and lysosomal-related genes and serves as a master regulator for lysosome biogenesis^34^. Here, we measured the transcriptional activity of TFEB in response to anlotinib treatment. In HEK293 cells transiently expressing GFP-TFEB, fluorescence microscopy analysis showed that anlotinib treatment increased the expression levels of TFEB and promoted the translocation of TFEB into the nucleus following 12 h of treatment (Fig. 5a). Consistently, we prepared cellular fractions from anlotinib-treated HCT116 cells and found that more TFEB accumulated in the nucleus after 12 h of anlotinib treatment (Fig. 5b). The translocation of TFEB into the nucleus suggests that the transcriptional activity of TFEB could be enhanced.

Under normal conditions, TFEB colocalizes with the master growth regulator mTOR on the lysosomal membrane and its activity is inhibited^16^. In anlotinib-treated cells, we previously confirmed that the mTOR signaling is inhibited (Fig. 4a). Thus, we performed an immunoprecipitation assay using FLAG to pull down both FLAG-TFEB and 14-3-3 and found that the interaction of TFEB with 14-3-3 decreased over time (Fig. 5c), suggesting a decrease in phospho-TFEB levels and the post-translational regulation of TFEB.

In addition, a TFEB promoter-driven luciferase reporter was used to measure the transcriptional activity of TFEB. As shown in Fig. 5d, anlotinib treatment significantly increased the luciferase activity of TFEB at the 12-h time point. Finally, we measured the levels of several known targets of TFEB, namely, ATP6V0E2 and CTSB (cathepsin B). In HCT116 cells, the mRNA levels of these genes were significantly increased following anlotinib treatment for 12 h (Fig. 5e). Taken together, these results suggest that anlotinib treatment enhances the transcriptional activity of TFEB and post-translational regulation of TFEB becomes the most important mechanism in lysosomal activation.

### Knockdown of TFEB or ATP6V0E2 impairs lysosomal activation by anlotinib

**Fig. 6:**
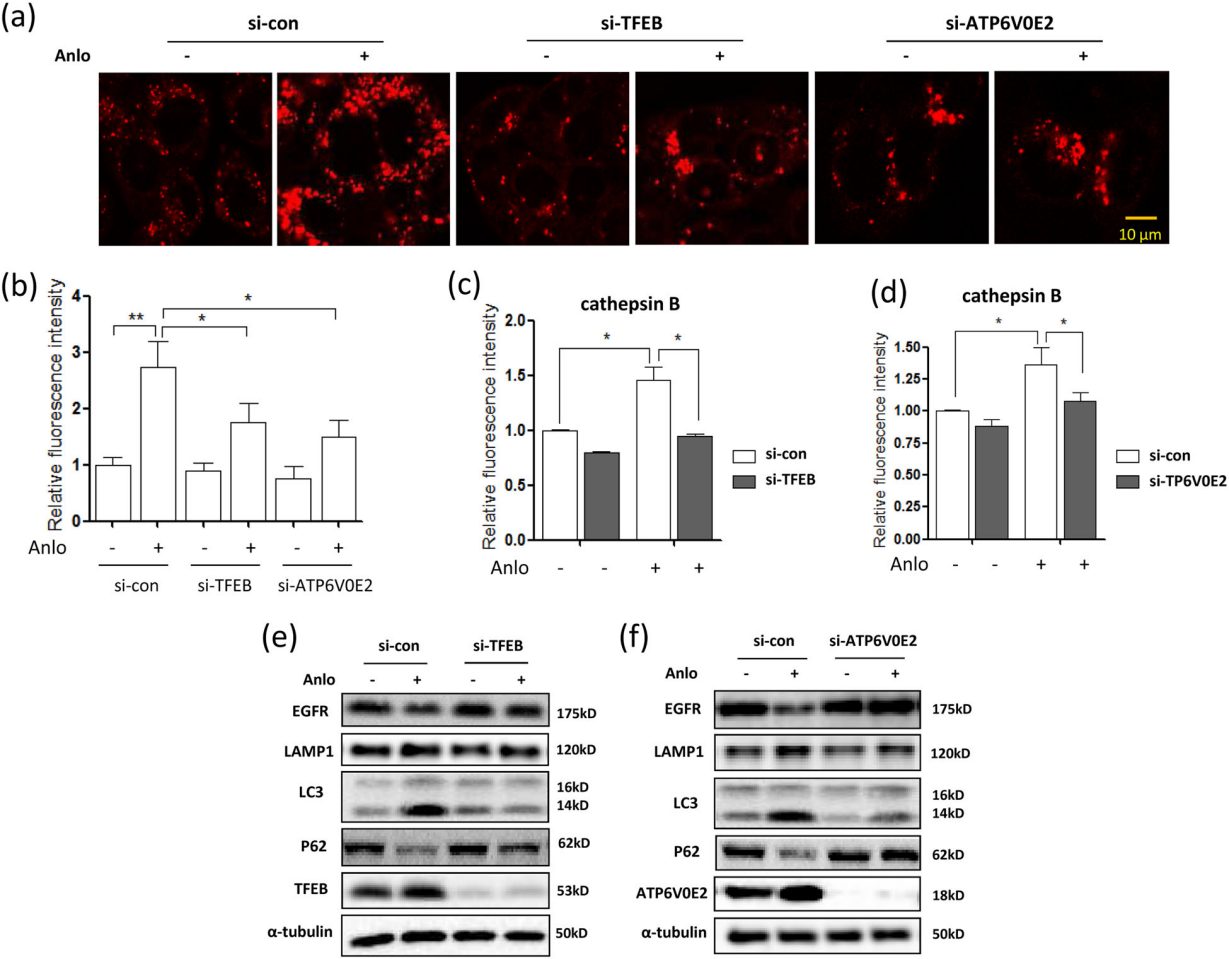
Knockdown of TFEB or ATP6V0E2 impairs lysosomal activation and autophagy induction by anlotinib. HCT116 cells were first transfected with siRNA for TFEB or ATP6V0E2 and then treated with anlotinib (2.5 μM) for 12 h. **a**, **b** LysoTracker staining was performed and examined by confocal microscopy (scale bar: 10 μm). Fluorescence intensity was calculated and statistically analyzed using Student’s *t*-test (*P < 0.05, **P < 0.01). **c**, **d** Magic Red for cathepsin B was loaded and measured by flow cytometry. The cell fluorescence values were calculated and statistically analyzed using Student’s *t*-test (*P < 0.05). **e**, **f** As in **a**, after the designated treatment, cells were harvested and lysed for western blotting. The levels of EGFR, LAMP1, LC3 and P62 were determined and α-tubulin was used as a loading control.

We next transiently knocked down TFEB or ATP6V0E2 to validate its regulatory role in anlotinib-mediated lysosomal activation. As shown in Fig. 6a, b, LysoTracker staining showed that TFEB or ATP6V0E2 knockdown decreased cell fluorescence in anlotinib-treated cells. In addition, the anlotinib-induced increase in lysosomal enzyme cathepsin B activity was attenuated by TFEB or ATP6V0E2 knockdown (Fig. 6c, d). Consistent with these result, the lysosomal degradation of EGFR induced by anlotinib was blocked in ATP6V0E2 or TFEB knockdown cells (Fig. 6e, f). Moreover, ATP6V0E2 or TFEB knockdown decreased autophagy levels in anlotinib-treated cells with little increase in LC3 levels or decrease in P62 levels (Fig. 6e, f). These results indicate that ATP6V0E2 and TFEB are required for anlotinib-induced lysosomal activation and autophagy induction.

### Lysosomal activation protects against cell apoptosis induced by anlotinib

**Fig. 7:**
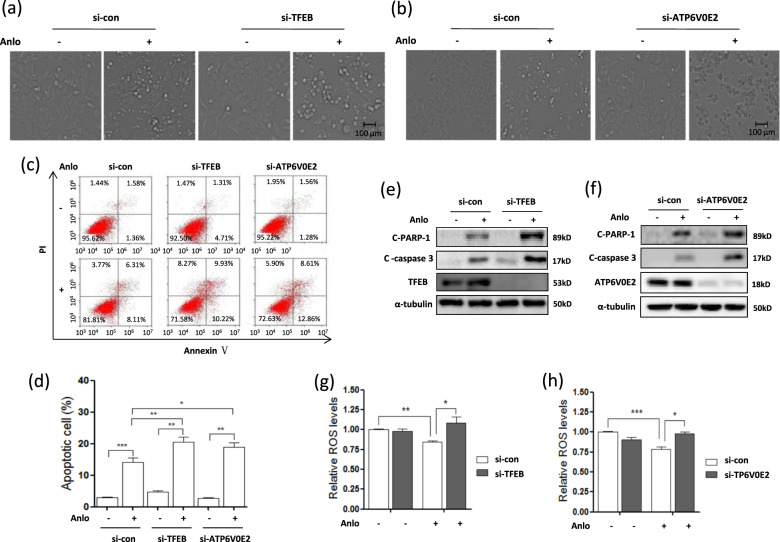
Inhibition of lysosomal function leads to more cell death by anlotinib. HCT116 cells were first transfected with siRNA for TFEB or ATP6V0E2 and then treated with anlotinib (5.0 μM) for 24 h. **a**, **b** Cell morphology changes were captured with an inverted microscope (scale bar: 100 μm). **c**, **d** The treated cells were stained with Annexin V-Pacific blue and PI and cell fluorescence was measured by flow cytometry. The data are presented as the mean ± SD (*P < 0.05, **P < 0.01, ***P < 0.001). **e**, **f** The treated cells were harvested and lysed for western blotting and cell apoptosis markers (cleaved PARP-1 and Caspase-3) were examined. **g**, **h** Cells with TFEB or ATP6V0E2 knockdown were treated with anlotinib (2.5 μM, 12 h) and then labeled with 1 μM CM-H_2_DCFDA for 10 min at 37 °C. Cellular fluorescence was measured using flow cytometry and statistically analyzed (*P < 0.05, **P < 0.01***, *P* < 0.001).

Next, we sought to examine the functional role of lysosomal activation in anlotinib-induced cytotoxicity. We knocked down TFEB or ATP6V0E2 expression in HCT116 cells and treated the cells with anlotinib. As shown in Fig. 7a, b, changes in cell morphology indicated that anlotinib treatment caused more cell death in TFEB or ATP6V0E2 knockdown cells. Next, we quantified the cell apoptosis using annexin V and PI staining^35^. The results showed that TFEB or ATP6V0E2 knockdown led to a significant increase in cell apoptosis upon anlotinib treatment (Fig. 7c, d and Supplementary Fig. 6). Consistently, western blotting results showed that the cleavage of Caspase-3 or PARP-1 representing cell apoptosis was markedly increased by anlotinib upon TFEB or ATP6V0E2 knockdown (Fig. 7e, f). The above findings demonstrate that lysosomal activation protects against anlotinib-induced cell apoptosis.

To further elucidate the link between lysosomal inhibition and cell apoptosis by anlotinib, we measured the changes in ROS levels in anlotinib-treated cells using a CM-H_2_DCFDA probe^36^. As shown in Fig. 7g, h, ATP6V0E2 knockdown significantly increased ROS levels in anlotinib-treated cells, and this increase may result in more cell death. These findings demonstrate that the lysosome protects against cell death by regulating the cellular redox status.

### Lysosomal inhibition enhances the tumor suppression by anlotinib

**Fig. 8:**
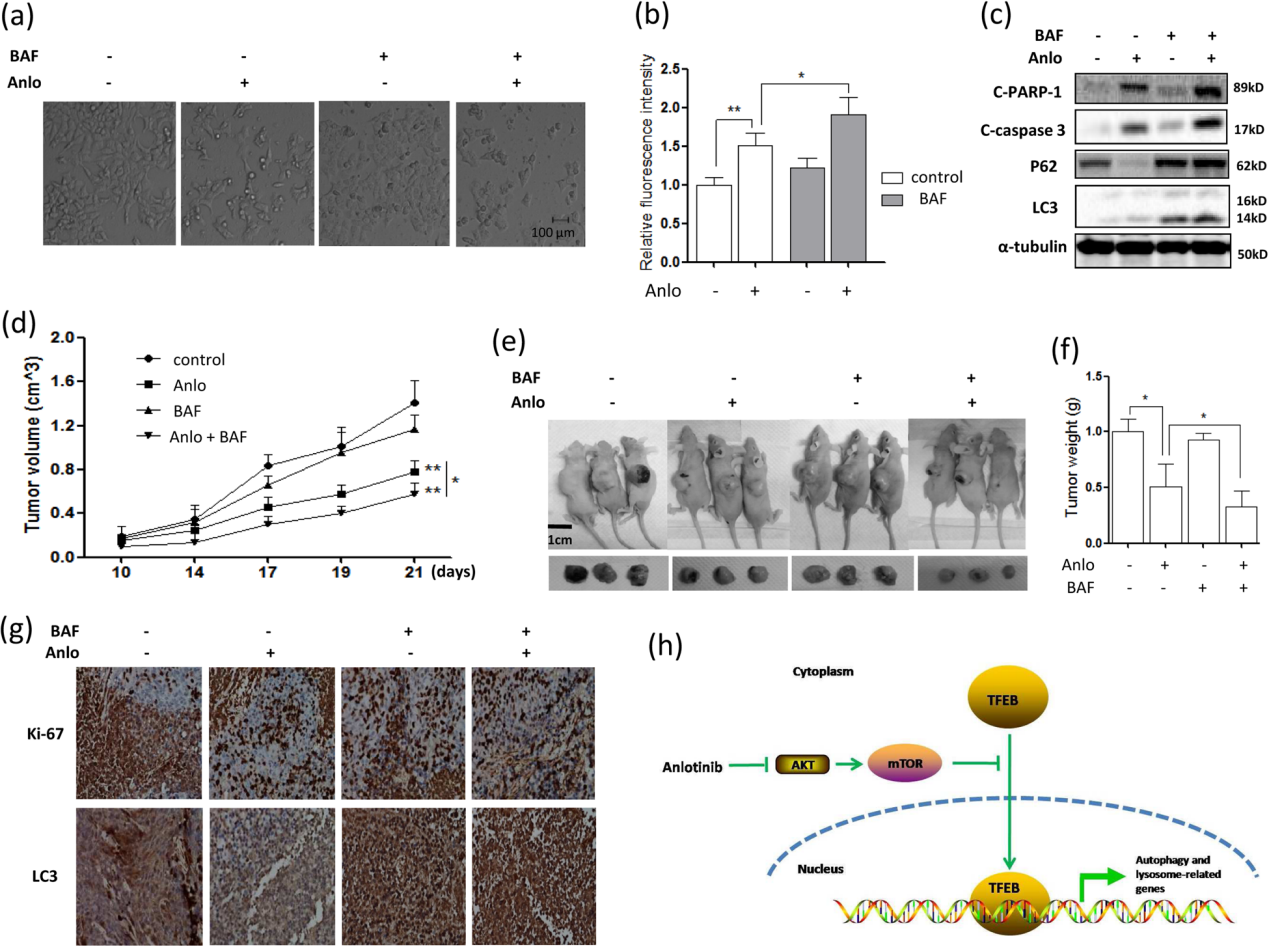
Inhibition of lysosomal function enhances the therapeutic efficacy of anlotinib. HCT116 cells were treated with anlotinib (5.0 μM, 24 h) in the presence of lysosomal inhibitor BAF (10 nM). **a** Cell morphology changes were captured with an inverted microscope (scale bar: 100 μm, up panel). **b** The treated cells were stained with Annexin V-Pacific blue and cell fluorescence was quantified by flow cytometry. The data are presented as the mean ± SD (*P < 0.05, **P < 0.01). **c** The treated cells were harvested and lysed for western blotting to measure the levels of cleaved PARP-1 and Caspase-3. Inhibition of tumor growth in vivo by anlotinib and BAF. **d** Xenograft tumor volumes were measured twice per week and then quantified and statistically analyzed. Anlotinib plus BAF had a more significant antitumor effect than anlotinib treatment alone; BAF alone treatment had little antitumor effect (*P < 0.05, **P < 0.01). **e** Typical images of the tumor-bearing mice with their xenograft tumors. **f** The average tumor weight for each group was calculated and statistically analyzed (*P < 0.05). **g** Representative images of IHC staining of Ki-67 and LC3 were performed on serial sections of tumors from various groups. **h** A schematic model of the activation of lysosomal function by anlotinib in human colon cancer.

Finally, we determined the lysosomal function in antitumor effect by anlotinib. We used lysosomal inhibitor BAF to determine the functional role of lysosome in anlotinib-induced cell death through blocking V-ATPase activity. Cell morphology changes also showed that anlotinib treatment induced more cell death under lysosomal inhibition (Fig. 8a). Consistently, autophagy inhibition significantly increased annexin V staining and the cleavages of caspase-3 and PARP-1 (Fig. 8b, c). These observations thus indicate that anlotinib-activated lysosomal function promotes cell survival.

Based on the above results, xenograft models were used to further confirm the effect of anlotinib-mediated autophagy and apoptosis on tumor growth in vivo. After HCT116 cells were injected subcutaneously into their right flanks, nude mice were divided into four groups. As shown in Fig. 8d, 10 days after inoculation, tumor-bearing mice were given an intratumoral injection of anlotinib with or without BAF, an inhibitor of vacuolar type H^+^-ATPase. No significant differences in tumor suppression were observed between the BAF-treated group and the PBS-treated control group. However, anlotinib treatment with or without BAF led to a significant tumor suppression in all nude mice (Fig. 8d). Moreover, compared to treatment with anlotinib alone, the combined treatment with anlotinib and BAF significantly suppressed tumor growth. At 21 days after inoculation, all mice were sacrificed and tumor was removed from mice. As expected, the tumor weight in the BAF treatment did not show a significant difference compared with those in the PBS control group (Fig. 8e, f). The combined treatment with anlotinib and BAF group exerted greater antitumor effect in HCT116 xenograft tumor models compared with the drugs administered independently. Moreover, the expressions of Ki-67 and LC3 in xenografts were detected by immunohistochemistry. Compared with any other group, the expressions of Ki-67 was significantly reduced in the combined treatment group (Fig. 8g), suggesting a lower proliferation rate. Accompaniedly, the levels of LC3 were significantly increased in the combined treatment group. These results indicate that lysosomal inhibition could enhance the anticancer effect of anlotinib in vivo.

## Discussion

Despite continued attempts to develop therapeutic and screening methods, colon cancer remains a major life-threatening malignancy. The common treatment is standard chemotherapy and targeted therapy followed by complete surgical resection^37^. Anlotinib is a novel oral multi-targeted receptor tyrosine kinase inhibitor that was synthesized to primarily inhibit a group of newly identified kinases^3^. In the present study, we sought to investigate the relationship between the lysosome and the therapeutic response to anlotinib in human colon cancer and further explore its possible mechanism, in an attempt to provide clinical therapy choices for colon cancer patients. Our findings demonstrate that the inhibition of lysosomal function can enhance the cytotoxicity of anlotinib in human colon cancer.

Previous studies showed that anti-angiogenesis agents can reduce tumor microvascular density and increase tumor hypoxia, leading to autophagy activation in tumor cells^38^. Here, in human colon cancer cells, we observed for the first time that autophagy flux is enhanced by anlotinib treatment, as indicated by an increase in LC3 (autophagosome marker) and a decrease in P62 (autophagy substrate) (Fig. 3a, b, e). Moreover, lysosomal functions are activated by anlotinib treatment, as evidenced by (i) an increase in lysosomal acidification (reduced lysosomal pH) (Fig. 3c, d); (ii) enhanced lysosomal cathepsin B and L activity (Fig. 4e); and (iii) increased lysosomal degradative activity EGFR degradation and GFP-LC3 cleavage (Fig. 3f, g). At the same time, we observed that cell apoptosis was also induced in anlotinib-treated cells with increasing time and dosage (Fig. 1). Recent studies suggest that the autophagy and apoptosis signaling pathways interact with each other^39–41^. The inhibition of autophagy function can enhance apoptosis in cancer cells^22,40^ but autophagy can also promote apoptosis in an inflammatory microenvironment^42^. In our present study, we demonstrated that adding lysosomal inhibitors such as BAF to block autophagy induces more cell growth inhibition and apoptosis induction by anlotinib (Fig. 8a–c). Although the function of autophagy induced by anti-angiogenesis agents remains ambiguous, it is considered to play a cytoprotective role in most cases.

At the late stage of autophagy, autophagosomes fuse with lysosomes and their contents are then degraded by the lysosomes^43^. We also determined the effect of anlotinib on this process and observed that anlotinib treatment enhances their fusions (Fig. 3h, i). It has been reported^27^ that the fusion of autophagosomes with lysosomes contributes to lysosomal activation, and we confirmed the positive effect of anlotinib on lysosomal function (Fig. 3). Due to mTOR suppression, the phosphorylation levels of TFEB was decreased accordingly (Fig. 5) and the post-translational regulation of TFEB became the most important mechanism regulating lysosomal function in anlotinib-treated cells. On the one hand, the lower phosphorylation levels of TFEB enhanced the transcriptional activity of TFEB^16,44^; on the other hand, the inhibition of the TFEB phosphorylation decreased its degradation and thus increased its expression levels (Fig. 5a)^45^. In the regulation of lysosomal function, a large number of lysosome-related genes were upregulated by anlotinib and ATP6V0E2 was first identified as a key target of anlotinib using transcriptome sequencing (Fig. 2c). It is one target of TFEB and transcriptionally regulated by the latter, which is the master regulator controlling lysosome biogenesis^17,34^. When ATP6V0E2 was knocked down, lysosomal activation was impaired in anlotinib-treated cells (Fig. 6), confirming its important function in lysosomal activation.

More recently^46,47^, lysosomes have been revealed to participate in tumor invasion, angiogenesis, metastasis and even some aspects of antiangiogenetic drug resistance. It satisfies the demand for metabolites by cancer cells and protects their survival in poor microenvironment through lysosomal degradation of cellular components^21^. In our study, when lysosomal functions were inhibited either by TFEB or ATP6V0E2 knockdown, anlotinib treatment increased cancer cell death (Figs. 7 and 8). Moreover, in vivo xenograft models of colon cancer showed that lysosomal inhibition by BAF enhances the tumor suppressive properties of anlotinib (Fig. 8d–f), suggesting that the lysosome serves to promote cell survival. Our results support the notion that anlotinib and lysosomal inhibition can function as a combined therapeutic strategy for enhanced antitumor therapy.

In the transcriptome sequencing, other targets related with apoptosis and oxidative stress pathways were also upregulated by anlotinib (Fig. 2c). The downstream targets of p53, CREBBP binds to CREB protein and involves in the transcriptional coactivation of many transcription factors^48^. Among its related pathways are direct p53 effectors^49^. Under oxidative stress by anlotinib, hypoxia increased the expression of SERPINE1 (ref. ^50^), one downstream target of HIF. Meanwhile, FOS proteins FOSB and FOSL1 (FOS like 1) also mediate transcriptional responses to oxidative stress by dimerizing with proteins of the JUN family to form AP-1 (activator protein 1) complex^51,52^. On the contrary, other targets involved in cell proliferation, protein trafficking and energy metabolism were downregulated (Fig. 2c). AKT1S1 (AKT1 substrate 1), a proline-rich substrate of AKT that binds 14-3-3 protein when phosphorylated^53^, regulating cell growth and survival, was downregulated under anlotinib treatment. The levels of several targets for protein trafficking were also decreased. SLC29A1 (solute carrier family 29 member 1), a transmembrane glycoprotein, serves as nucleoside transporters^54^. Vps25 (vacuolar protein sorting 25 homolog) and Vps72 (vacuolar protein sorting 72 homolog) play a role in vesicle-mediated protein trafficking^55,56^. Flot2 (Flotillin 2) is a caveolae-associated, integral membrane protein involved in vesicular trafficking and signal transduction^57^. Meanwhile, a series of targets, which encode mitochondrial enzyme and involve in energy metabolism, were also significantly downregulated and failed to protect cells against oxidative damage. GPX1 (glutathione peroxidase 1) catalyzes the reduction of organic hydroperoxides and hydrogen peroxide (H_2_O_2_) by glutathione^58^. GPD1L (glycerol-3-phosphate dehydrogenase 1 like) plays a critical role in carbohydrate and lipid metabolism^59^. ALDH3B1 (aldehyde dehydrogenase) enzymes are critical in the detoxification of aldehydes^60^. In addition, the levels of genes encoding mitochondrial membrane protein to maintain membrane integrity were also decreased. TIMM10 (translocase of inner mitochondrial membrane 10) is an essential protein of the mitochondrial intermembrane space^61^. FIS1 (fission, mitochondrial 1) regulates mitochondrial morphology^62^. The above results indicated that under anlotinib treatment, cells growth and metabolism slow down and demonstrate more stress responses. Thus, we speculate that anlotinib may exert its biological function through various different pathways.

Taken together, our findings demonstrate a novel mechanism of the anticancer drug anlotinib in human colon cancer in which lysosomal function is activated to protect against cell apoptosis (Fig. 8h). Thus, the inhibition of lysosomal function can be developed as a novel method to increase the cytotoxicity of anlotinib.

## Supplementary information

Suppl Figure legends

Suppl Figure 1

Suppl Figure 2

Suppl Figure 3

Suppl Figure 4

Suppl Figure 5

Suppl Figure 6

Suppl Figure 7

Suppl table 1

Suppl table 2

Suppl table 3
