# Supplementary material for: Transcriptome profiling analysis reveals that ATP6V0E2 is involved in the lysosomal activation by anlotinib
Source: Cell Death Dis. 2020 Aug 24;11(8):702. doi: 10.1038/s41419-020-02904-0 (PMC7445181; doi:10.1038/s41419-020-02904-0)
Supplement: Supplementary file 1 — Suppl Figure legends [file 41419_2020_2904_MOESM1_ESM.docx]

**Supplementary Figure legends**

**SF. 1 Cell proliferation inhibition was determined by colony formation assays in human colon cancer cells.** **a,b** HCT116 or SW480 cells were treated with various concentrations of anlotinib (1 or 2.5 μM). **c,d** The number of clonies were counted by Image J and the statistical analysis was shown in the bar diagram for three individual experiments in HCT116 and SW480 cells, respectively. The asterisks indicate statistical significance (* *P* < 0.05, ***P* < 0.01).

**SF. 2 Anlotinib induces apoptosis in human colon cancer cells. a** HCT116 and SW480 cells were treated with anlotinib (1~10 μM) for 24 hours as indicated. Cells were stained using Annexin V-Pacific blue and PI, and cell fluorescence was detected with flow cytometry. Scatter blots were shown. **b** as in **a**, cell fluorescence intensity of Annexin V staining was calculated and statistically analysed. **c** HCT116 and SW480 cells were treated with 5.0 μM anlotinib for different times (6, 12 or 24 hours) as indicated. After Annexin V staining, cell fluorescence was quantified using flow cytometry. Statistical analysis was performed using Student’s *t* test (**P* < 0.05 ***P* < 0.01).

**SF. 3 The signaling pathways with related genes in anlotinib-treated cells after KEGG enrichment.** FC fold change.

**SF. 4 Effect of anlotinib treatment on the autophagy in human colon cancer cells.** SW480 cells were treated with anlotinib (2.5 μM) with or without bafilomycin for 12 hours. The cells were then harvested for western blotting to examine LC3 and P62 levels. β-actin served as a loading control.

**SF. 5 Anlotinib treatment inhibits mTOR signaling.** SW480 cells were treated with different doses of anlotinib (1.0, 2.5 or 5 µM, left panel) or treated with anlotinib (2.5 µM) for different times (6, 12 or 24 hours, right panel) as indicated. Cells were harvested and lysed for western blotting to determine phospho-AKT (Ser473) and phospho-S6 (Ser235/236) levels. β-actin was used as a loading control.

**SF. 6 Inhibition of lysosomal function leads to more cell death by anlotinib.** HCT116 cells were first transfected with siRNA for TFEB or ATP6V0E2 and then treated with anlotinib (5.0 μM) for 24 hours. **a,b** The treated cells were stained with Annexin V-Pacific blue and cell fluorescence was measured by flow cytometry. The data are presented as the mean ± SD (* *P* < 0.05, ***P* < 0.01).

**SF. 7 Measurement of cell viability under anlotinib treatment alone or with bafilomycin.** HCT116 cells were treated with anlotinib (1.0 µM, 2.5 µM, 5.0 µM, 7.5 µM; 24 hours) with or without lysosomal inhibitor BAF (10 nM). **a** The treated cells were stained with PI and cell death percentage was quantified by flow cytometry. The data are presented as the mean ± SD. **b** Scatter plots of different treatments were shown from flow cytometry. **c** as in a, after indicated treatment, 10 μl of CCK-8 solution was add into each well of the plate and the plate was incubated for 1 hour in the incubator. The absorbance at 450 nm was measured using a microplate reader and analysed.

**Supplementary Table legends**

**STable 1. GO analysis of cellular localization of the anlotinib targets.**

**STable 2. KEGG enrichment of signaling pathways of the anlotinib targets.**

**STable 3. Expression level changes of the anlotinib target genes.**
